# Characteristics of Multifunctional, Eco-Friendly Lignin-Al_2_O_3_ Hybrid Fillers and Their Influence on the Properties of Composites for Abrasive Tools

**DOI:** 10.3390/molecules22111920

**Published:** 2017-11-07

**Authors:** Łukasz Klapiszewski, Artur Jamrozik, Beata Strzemiecka, Iwona Koltsov, Bartłomiej Borek, Danuta Matykiewicz, Adam Voelkel, Teofil Jesionowski

**Affiliations:** 1 Institute of Chemical Technology and Engineering, Faculty of Chemical Technology, Poznan University of Technology, Berdychowo 4, PL-60965 Poznan, Poland; artur.robert.jamrozik@gmail.com (A.J.); beata.strzemiecka@put.poznan.pl (B.S.); adam.voelkel@put.poznan.pl (A.V.); teofil.jesionowski@put.poznan.pl (T.J.); 2 Wielkopolska Centre of Advanced Technologies, Umultowska 89 C, PL-61614 Poznan, Poland; 3 Polish Academy of Sciences, Institute of High Pressure Physics, Sokolowska 29/37, PL-01142 Warszawa, Poland; iwona.koltsov@gmail.com; 4 RHL-Service, Budziszynska 74, PL-60179 Poznan, Poland; bartek@rhl.pl; 5 Institute of Materials Technology, Faculty of Mechanical Engineering and Management, Poznan University of Technology, Piotrowo 3, PL-61138 Poznan, Poland; danuta.matykiewicz@put.poznan.pl

**Keywords:** lignin-Al_2_O_3_ hybrid materials, abrasive tools, lignin, thermomechanical properties, rheological studies

## Abstract

The main aim of the present study was the preparation and comprehensive characterization of innovative additives to abrasive materials based on functional, pro-ecological lignin-alumina hybrid fillers. The behavior of lignin, alumina and lignin-Al_2_O_3_ hybrids in a resin matrix was explained on the basis of their surface and application properties determined by inverse gas chromatography, the degree of adhesion/cohesion between components, thermomechanical and rheological properties. On the basis of the presented results, a hypothetical mechanism of interactions between lignin and Al_2_O_3_ as well as between lignin-Al_2_O_3_ hybrids and phenolic resins was proposed. It was concluded that lignin compounds can provide new, promising properties for a phenolic binder combining the good properties of this biopolymer as a plasticizer and of alumina as a filler improving mechanical and thermal properties. The use of such materials may be relatively non-complicated and efficient way to improve the performance of bonded abrasive tools.

## 1. Introduction

Resin-bonded abrasive products are complex composites consisting of abrasive, wetting agent (e.g., resole), binder (e.g., novolac), and fillers (e.g., pyrite, cryolite) [1]. Several factors influence the properties of the final abrasive tool during the production process and exploitation. The first stage during production of abrasive tools is covering the abrasive grains by resole. Appropriate covering of grains by resole is crucial for homogeneity of the semi-product and the final product [2]. In the next stage novolac mixed with filler is added. Then, the semi-product is pressed and hardened according to a specific temperature program. The appropriate hardening of the semi-product is highly important for the efficiency of the final product [3]. Hardening of resins in the final product also depends on the fillers used. Some of them can accelerate the hardening rate and some of them can modify this process [3]. Moreover, the fillers play a very important role in the work of the grinding tools, as they collect heat and prevent melting of the resin [4,5]. Inorganic compounds are broadly used as fillers. Conjugation of these fillers, characterized by polar surface properties, with a non-polar polymer matrix is difficult [6]. The use of an organic-inorganic hybrid filler may overcome this problem. Moreover, such hybrid fillers may increase the thermal resistance and mechanical strength. This effect may result from the possible reactions between active groups present in the inorganic and organic components.

Alumina is one of the most commonly used abrasive materials. In the present study, alumina was used as a filler. An Al_2_O_3_ filler can act as an additional abrasive and can collect heat. In order to increase the functionality of the final product, alumina was combined with lignin. Lignin is a natural polymer with a similar structure to phenolic resins used as binders in abrasive articles. Today, interest in natural resource polymers is growing due to the depletion of conventional petrochemical resources [7]. There are already known cases of successful use of biopolymers such as cellulose in advanced applications [8,9]. Lignin is the most available material in nature after cellulose [10]. Modified lignin is a polarographically active material and in recent years this biopolymer has also found interesting applications in electrochemistry [11,12,13,14]. As an aromatic biopolymer, it is a potential substitute for the polymers obtained from petroleum, due to its comparable or improved physicochemical properties and lower manufacturing cost. The presence of numerous hydroxyl groups in aromatic rings enables its use as a starting material for the synthesis of a wide range of polymers (such as polyethers, polyesters, polyethylene and polyurethane) [15]. Literature reports also suggest the potential use of lignocellulosic materials, including pure lignin and/or lignosulfonate, as fillers in a large group of polymers [16,17,18,19,20,21,22]. The problem of application of lignin to polyolefins was described in [19,20,21,22]. In case of mixing lignin with phenolic resins, the problem is not associated with the homogeneity of the polymer-lignin system, but insufficient mechanical properties [23,24]. Thus, it is expected that the application of a lignin-alumina hybrid as a filler may improve the mechanical properties of the final product. A very important aspect of the use of lignin-alumina hybrid as a filler is the reduction of emissions of harmful compounds into the atmosphere, due to the increased thermal stability of such a system in comparison with phenolic resins and/or lignin systems [24]. The described biopolymer is also one of the potential low-cost and readily available sorbents of environmentally harmful metal ions [25,26]. In order to be used as a sorbent, lignin can be obtained chiefly as a waste product from the paper industry and subjected to chemical modification to increase the number of functional groups [27].

There is a limited number of reports which describe attempts to use lignin and/or lignosulfonate in the preparation of advanced inorganic-organic hybrid materials. The concern is mainly the combination of biopolymers with the widely used and well-established silica [13,14,21,22,26,28,29,30]. Direct linking of natural polymers (lignin and lignosulfonates) with alumina has not been previously described.

The aim of our study was the preparation of new hybrid lignin-alumina fillers, which have not yet been described in the literature. The next step will be to test applications of the model composites in the abrasive industry. Lignin-alumina hybrid fillers were preliminarily tested to establish whether they may serve as new, promising, eco-friendly fillers for abrasive tool production. It is expected that such hybrid fillers should: (i) reinforce the final composite and (ii) possess higher thermal stability than lignin itself.

## 2. Results

### 2.1. Dispersive-Morphological Properties of Lignin-Al_2_O_3_ Hybrids

**Figure 1:**
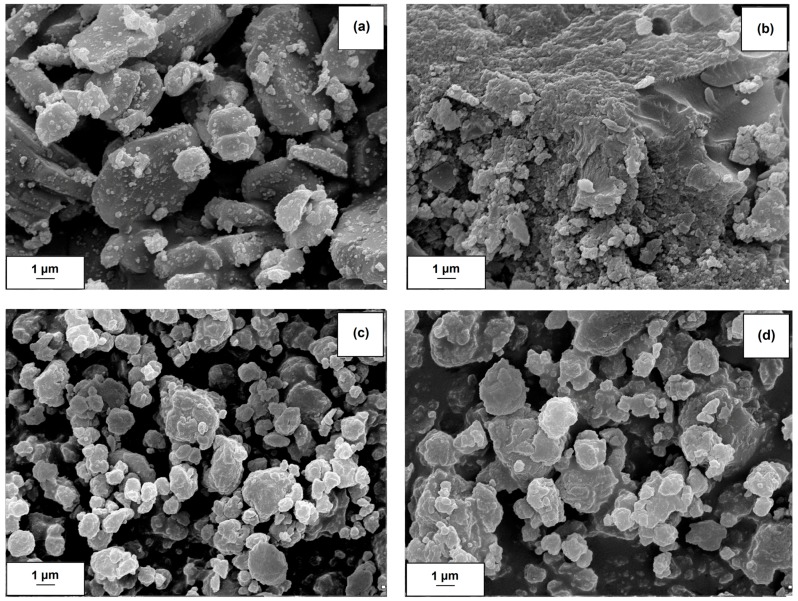
SEM images of alumina (**a**); lignin (**b**) and lignin-alumina hybrid materials (8:1, *wt*/*wt*) (**c**) and (8:6, *wt*/*wt*) (**d**).

Aluminum oxide exhibited the presence of primary particles with diameters close to 100 nm, which showed a tendency to form aggregates (<1 µm). Al_2_O_3_ had different dispersive-morphological properties (see Table 1 and Figure 1a).

The particle size distribution of Al_2_O_3_ is very broad (from 142 nm to 955 nm, data from a Zetasizer Nano ZS apparatus). Addition of lignin slightly increased the particle size distribution. As follows from the data presented in Table 1, the increased lignin content in the hybrid filler resulted in a shift of the size distribution of particles (including primary particles and agglomerates, respectively) to larger sizes. It should be noted that the commercial Kraft lignin used in the study contains particles of a wide range of sizes, which indicates the possibility to form large agglomerate structures. The presence of primary particles and secondary agglomerates was also confirmed by SEM images. Figure 1a,b present the SEM images of Al_2_O_3_ and lignin, respectively, while Figure 1c,d show images of lignin-alumina hybrids obtained with the use of different ratios of lignin to Al_2_O_3_ (8:1 *wt*/*wt* and 8:6 *wt*/*wt* respectively). It can be observed that 50% by volume of the lignin-alumina (8:1 *wt*/*wt*) hybrid system was occupied by particles with diameters smaller than 3.6 μm, while 90% of the sample volume was taken up by particles with diameters smaller than 5.3 μm. The average particle size in the hybrid system was 3.3 μm (see Table 1).

### 2.2. Fourier Transform Infrared Spectroscopy

**Figure 2:**
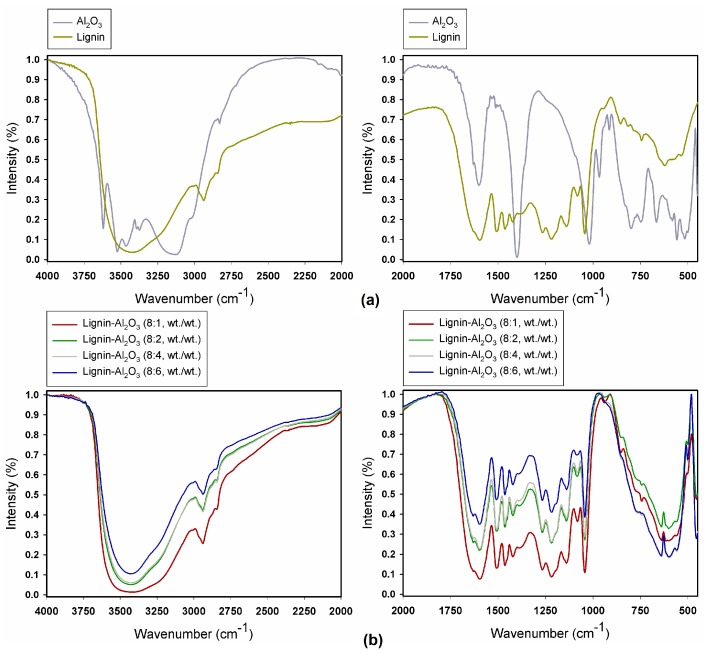
FTIR spectra of pure alumina and lignin (**a**) and of lignin-Al_2_O_3_ hybrid fillers (**b**).

FTIR analysis was performed in order to identify the functional groups present in the structure of alumina, lignin (Figure 2a) and lignin-Al_2_O_3_ hybrid fillers (Figure 2b). The most important bands are summarized in Table 2.

The spectrum obtained for pure alumina (Figure 2a) revealed the presence of physically bound water, confirmed by the band at 3145 cm^−1^, which results from O–H group stretching vibrations. Additionally, the band at 1620 cm^−1^ is caused by bending vibrations of the same group [31]. The bands at 3635 cm^−1^, 3543 cm^−1^ and 3473 cm^−1^ are attributed to Al–OH stretching vibrations [32]. Symmetric bending vibrations of Al–OH produce a band at 1035 cm^−1^, while the bands at 788 cm^−1^, 750 cm^−1^, 693 cm^−1^, 564 cm^−1^ and 512 cm^−1^ are attributed to Al–O vibrations, in which aluminum ions occupy both tetrahedral and octahedral sites [33].

Figure 2a also shows the spectrum of pure lignin. The results show the presence of stretching vibrations of O–H groups (phenolic O–H and aliphatic O–H) at 3432 cm^−1^, and stretching vibrations of C–H (–CH_2_ and –CH_3_) at 2965–2830 cm^−1^. Stretching vibrations from ketone groups (C=O) are associated with the band at 1648 cm^−1^, while those at 1602 cm^−1^, 1508 cm^−1^ and 1419 cm^−1^ are attributed to stretching vibrations of the C–C, C=C bonds in the aromatic skeleton. Stretching vibrations of ether groups (C–O–C) appear at 1095–1000 cm^−1^, and further bands in the range 1345–1250 cm^−1^ correspond to C–O stretching vibrations (C–O(H), C–O(Ar)). Below a value of 1000 cm^−1^ the spectrum contains bands attributed to in-plane and out-of-plane bending vibrations of aromatic C–H bonds. These results are in full agreement with earlier work [29,30,34,35].

The FTIR spectra of lignin-Al_2_O_3_ hybrid materials are presented in Figure 2b. The spectra revealed the presence of characteristic bonds for alumina: Al–O stretching vibrations at 1389 cm^−1^ (Al–O as Si cage (TO_4_)) and Al–OH symmetric bending vibrations at 1039 cm^−1^. The bands at 788 cm^−1^, 751 cm^−1^, 695 cm^−1^, 565 cm^−1^ and 512 cm^−1^ are attributed to bending vibrations of Al–O. An important broad band in the range 3600–3200 cm^−1^ comes from stretching vibrations of O–H groups, which occur in the structure of both lignin and alumina. Functional groups which were observed in pure lignin are also present: C–H bonds at 2937 cm^−1^ and 2879 cm^−1^, and different types of carbon atom bonds in the 1650–1000 cm^−1^ range.

Based on the FTIR spectra for the pure precursors (alumina and lignin) and organic-inorganic hybrid fillers, it can be observed that the intensity of bands in the hybrids increased in comparison with pure materials. This confirms the effectiveness of the proposed method of preparation. It is associated with an increase in the intensity of the bands attributed to particular functional groups. Furthermore, the intensity of the bands increased with increasing content of lignin relative to alumina.

### 2.3. Thermogravimetric Analysis–Mass Spectrometry

**Figure 3:**
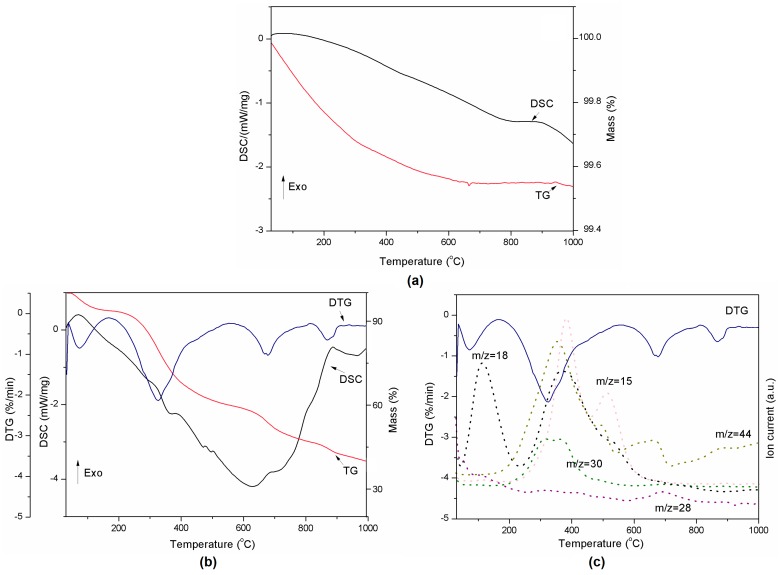
Thermal decomposition of Al_2_O_3_ represented by DSC-TG curves (**a**) and thermal decomposition of lignin represented by DSC-TG-DTG (**b**) and DTG-MS curves (**c**).

**Figure 4:**
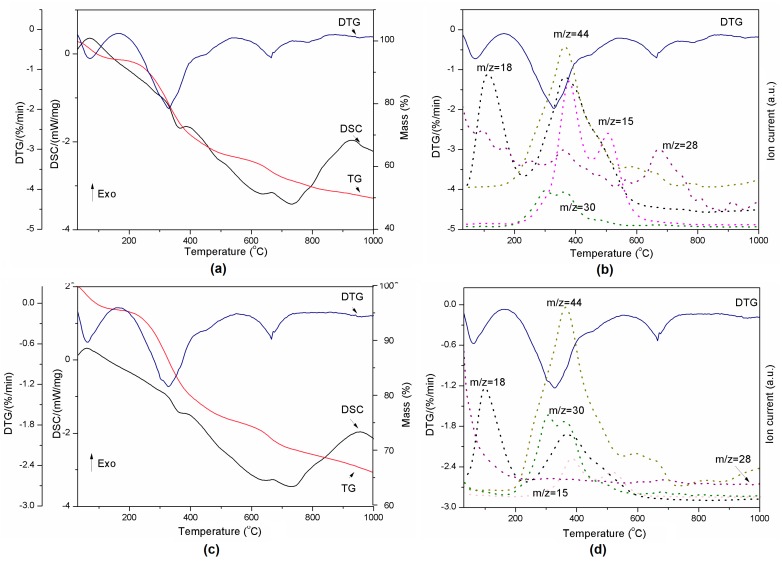
Thermal decomposition of lignin-Al_2_O_3_ (8:1, *wt*/*wt*) represented by DSC-TG-DTG (**a**) and DTG-MS curves (**b**) and lignin-Al_2_O_3_ (8:6, *wt*/*wt*) represented by DSC-TG-DTG (**c**) and DTG-MS curves (**d**).

**Figure 5:**
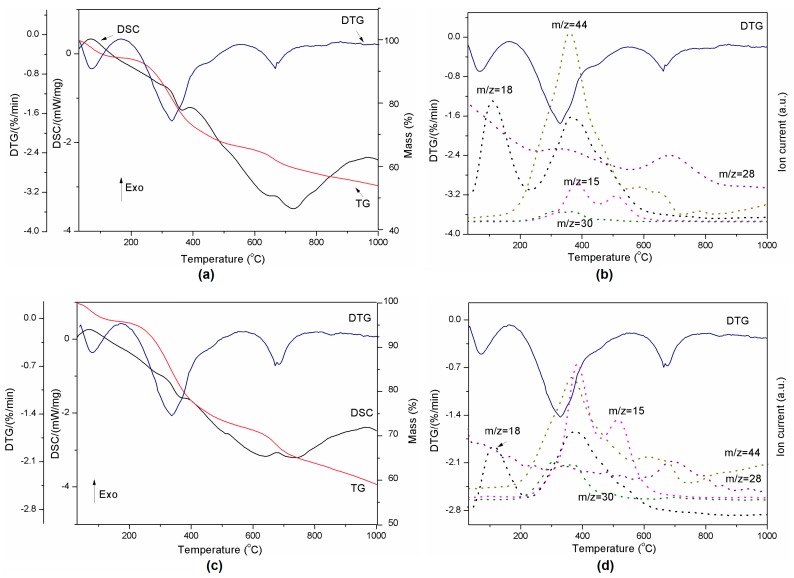
Thermal decomposition of lignin-Al_2_O_3_ (8:2, *wt*/*wt*) represented by DSC-TG-DTG (**a**) and DTG-MS curves (**b**) and lignin-Al_2_O_3_ (8:4, *wt*/*wt*) represented by DSC-TG-DTG (**c**) and DTG-MS curves (**d**).

The thermal decomposition of the pure components is presented in Figure 3a,b. The alumina used in the preparation of lignin composites did not show any transition in the DSC curve during thermal treatment (Figure 3a). However, the decomposition of pure lignin produced four events which are clearly visible on the TG and DTG curves (Figure 3b). Degradation of lignin is influenced by its nature and by the reaction temperature, heating rate and degradation atmosphere [36]. Sample mass loss while heating occurs due to release of water (between RT and 200 °C) and other lignin decomposition products such as CH_3_ (*m*/*z* = 15), CO (*m*/*z* = 28), HCHO (*m*/*z* = 30), and CO_2_ (*m*/*z* = 44) (see Figure 3c) [37]. The decomposition of the polymer structure in lignin begins at 200 °C and continues up to 700 °C. These observations are in agreement with [36]. The DSC-TG-MS results for all compositions presented in Figure 4 and Figure 5 show that there is no difference in terms of the trends of curves between samples. However, DTG curves for lignin-Al_2_O_3_ composites show lack of signal above 800 °C characteristic for pure lignin and CO_2_ release.

The presence of Al_2_O_3_ in a composite slightly reduced the onset temperature of H_2_O release from the material. This fact is visible especially in case of compositions from 8:2 *wt*/*wt* to 8:6 *wt*/*wt* (Table 3). The second transition started at higher temperatures for all composites than for pure lignin. This confirms that such material is more stable than Kraft lignin at the temperatures under 200 °C, which are most common for the grinding process in the presence of coolants. The third endothermic event related to CO_2_/N_2_ release varied between compositions and the highest onset temperature was found for the 8:2 mixture. The decomposition rate of materials presented in Table 3 increased with Al_2_O_3_ amount. The exception was composition 8:6 *wt*/*wt*, probably due to the smallest amount of sample mass loss.

In addition, the remaining results of thermal analysis are presented in Figure 5 and Table 3. All powders released the same gases as pure lignin during heating. However, in contrast to pure lignin, the lignin-alumina samples produced only three events during heating, at ~30, ~325 and ~650 °C. They did not exhibit a transition at 865 °C. The results indicate that the increase of Al_2_O_3_ quantity slows down the reaction at approximately 325 °C (see Table 3), when the gases are released.

### 2.4. Inverse Gas Chromatography

IGC analysis was used to evaluate the surface properties of the fillers (see Table 4). All of the studied materials demonstrated medium surface activity ($\gamma_{s}^{d}$ about 35–40 mJ/m^2^). Such values of $\gamma_{s}^{d}$ are in agreement with data published in the literature, e.g., for phenol-formaldehyde-lignin resin (41.9 mJ/m^2^ for a system with 17% of lignin in place of phenol) [38]. Organic-inorganic hybrid fillers with higher lignin content exhibit similar surface properties to lignin, but they are slightly less active, as some active groups may be connected to hydroxyl groups on the aluminum oxide surface.

This is in agreement with the FITR spectra, where it can be seen that the signal from OH groups decreases for lignin-Al_2_O_3_ hybrids compared to pure lignin (see Figure 2) and OH groups for Al_2_O_3_ are not visible for hybrid fillers. Interestingly, the hybrid fillers with the highest amount of alumina have acid-base surface properties similar to those of alumina (products have a similar agglomeration behavior). Thus, a hybrid filler with a lignin-to-alumina ratio of 8:6 *wt*/*wt* has different surface properties than the other studied hybrid materials, and can behave differently in the abrasive article. It is reflected for example in the different rheological properties for composite with a lignin-to-Al_2_O_3_ ratio of 8:6 *wt*/*wt* (Table 5). Moreover, this is in agreement with particle size distribution results: the hybrid with a lignin-to-Al_2_O_3_ ratio of 8:6 was characterized by a similar size distribution to that of alumina (see Section 2.1).

**Figure 6:**
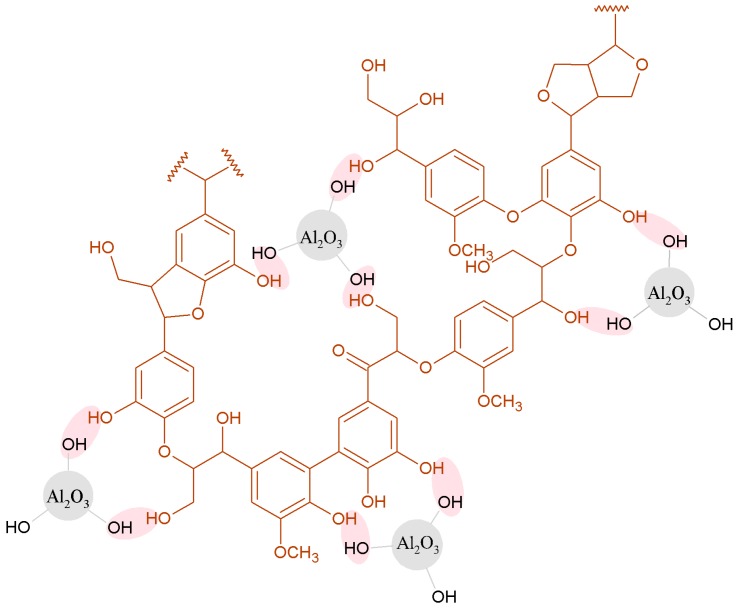
Hypothetical interactions between lignin and alumina.

All of the studied fillers are more likely to act as electron donors than acceptors. As regards the Al_2_O_3_ surface, O atoms with a free electron pair can act as electron donors, and the higher value of *K_D_* than *K_A_* indicates that the access of the test compounds to O atoms on the alumina surface is easier than to Al atoms with an electron gap. In the case of the hybrid fillers, the values of the *K_A_* and *K_D_* parameters are lower, as some active groups are involved in the linking between lignin and alumina. In the case of the hybrid with a lignin-to-silica ratio of 8:6 *wt*/*wt*, the surface has acid-base properties similar to those of alumina. The hypothetical interactions between lignin and alumina are presented in Figure 6.

### 2.5. Rheological Studies

All samples after the first preparation steps still had a powder form after rotation. For the 8:1 and 8:2 *wt*/*wt* lignin-alumina hybrid samples, a problem was encountered in reaching a measuring gap of 1.2 mm. For these samples the maximum normal force achieved was 50 N, because they had almost two times lower bulk densities and different thermal conductivity than the other samples. It is possible that the samples were not sufficiently softened. When the first step of sample preparation is analyzed (in terms of the relative change of the gap in time/temperature), conclusions can be drawn about the softening process, namely if the gap starts changing earlier (at lower temperature), the studied sample has a lower softening point; for example, for pure resin it is 84.2 °C, but for the resin with lignin-alumina additive (8:1, *wt*/*wt*) it is 86.4 °C. The relative change of the gap from the initial position provides information about the degree of softening and partly about thermal conductivity. For a larger relative change, it can be assumed that the sample is more plastic; e.g., for pure resin the size of the relative change of gap is 0.37 mm, while for the resin with the 8:6 *wt*/*wt* additive the change is 0.15 mm.

When it is observed, the G′ and G″ results for the pure novolac, 9% + lignin and 9% alumina samples in the first preparation steps are very noisy. This is caused by the pure contact of the rotor with the sample. For the pure novolac and 9% + alumina samples, it is noticed that the normal force is very low—which means weak contact with the rotor, because the volume of the samples changes during the softening process. For the 9% + lignin sample a big force it is obtained—this means weak contact with the rotor, because the sample is still a powder and slippage effects are observed. These conditions can’t be changed because the 1.2 mm gap should be constant and it is a compromise between a liquid sample and a solid.

During the experimental curing process, the pure resin is always in liquid state, even after 15 min at 160 °C, as it is thermoplastic without the addition of a cross-linking agent (urotropine). The other samples were solids at the end of the measurements. Table 5 summarizes the characteristic points for the curing process. The softening point was defined as the lowest value of the complex viscosity. At this point the sample has its most liquid form. After this point the curing process begins, where the storage modulus G′ and loss modulus G″ increase. The second characteristic point is the crossover with the same value G′ = G″. Prior to this crossover point, the measured material acts as a fluid more than elastic solid; afterwards storage modulus starts growing up faster than loss modulus and material acts as an elastic solid more than fluid.

**Figure 7:**
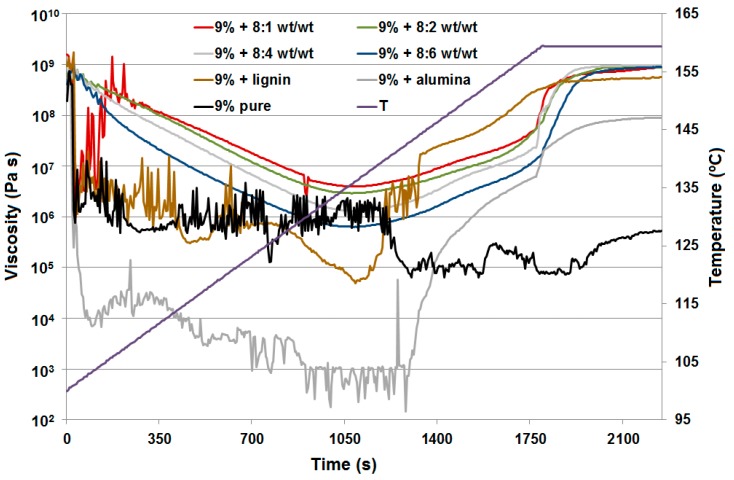
The curing process as complex viscosity.

**Figure 8:**
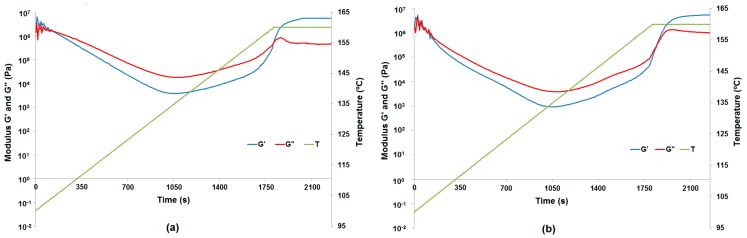
Phase transformation of lignin-Al_2_O_3_ (8:2, *wt*/*wt*) represented by the moduli G′ and G″ (**a**) and phase transformation of lignin-Al_2_O_3_ (8:6, *wt*/*wt*) represented by the moduli G′ and G″ (**b**).

For four lignin-alumina samples (8:1, 8:4, 8:2 and 8:6 *wt*/*wt*) the modulus of complex viscosity was at the same level at the end of the measurements. The results of the curing process are presented in Figure 7 as complex viscosity at measuring points, to show the influence of the additives on the curing process. Examples of phase transformation are shown in Figure 8a for resole with the 8:2 *wt*/*wt* lignin-alumina hybrid, and in Figure 8b for resole with the 8:6 *wt*/*wt* hybrid.

**Figure 9:**
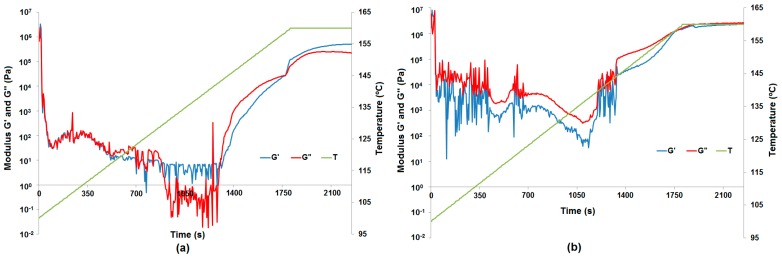
Phase transformation of resin with pure Al_2_O_3_ represented by the moduli G′ and G″ (**a**) and phase transformation of resin with pure lignin represented by the moduli G′ and G″ (**b**).

At the beginning of the curing process up to 104 °C, the samples of all hybrid materials (8:1, 8:2, 8:4 and 8:6 *wt*/*wt*) were more elastic solids (powder-like) because the storage modulus G′ is greater than the loss modulus G″. Next, a softening process was observed, where the sample acted as a fluid more than an elastic solid up to a temperature of 136 °C. After that the curing process starts, but the result differ depending on the rate of temperature change. For pure resin and resin with the addition of Al_2_O_3_ (Figure 9a), lignin-Al_2_O_3_ hybrid material (8:6 *wt*/*wt*) and pure lignin (Figure 9b), the measurements up to 140 °C appear disrupted because the softening process was different. Additionally, an increase in the normal force at the measuring point is observed in the graph above 140 °C. The sample expanded, the normal force increased, and the measurement conditions were better to preserve the moduli G′ and G″. For all samples apart from pure resin, a characteristic change in normal force was observed. The range of change in the normal force provides information about the internal dynamic process: if the force is greater, the process is more turbulent. For the resin with pure lignin, the normal force reached 15 N, but it was lower for systems with lignin-Al_2_O_3_ additives (8:1, 8:2, 8:4 and 8:6 *wt*/*wt*), and reached the lowest value (1.6 N) for systems with Al_2_O_3_.

### 2.6. Dynamic-Mechanical Properties

**Figure 10:**
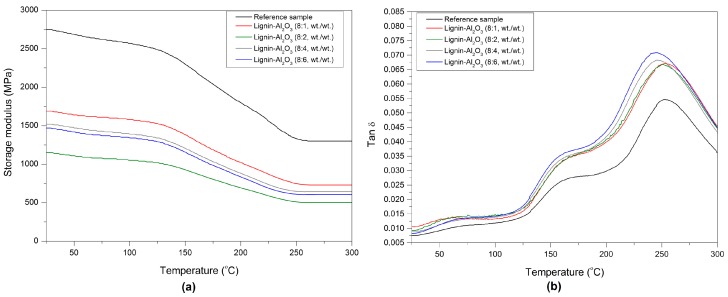
Storage modulus G′ (**a**) and tan δ (**b**) versus temperature for the composites obtained.

DMTA analysis is often used to assess the interaction between materials and provides information about the viscoelastic behavior of the composites, described by the storage modulus G′ and glass transition temperature Tg [39,40,41]. The values of Tg and G′ for the composites determined at various temperatures are given in Table 6. The glass transition temperature Tg is described as a single number representing a wide temperature region. The position of tan δ at its maximum was taken as the glass transition temperature of the composites [42]. Plots of the storage modulus (G′) and mechanical loss factor tan δ versus temperature T are shown in Figure 10a,b. The G′ values of the modified composites decrease with an increase in the lignin-Al_2_O_3_ content. The highest value of G′ (2750 MPa) was observed for the reference sample. The sample with lignin-Al_2_O_3_ (8:1, *wt*/*wt*) gave the highest value of G′ among the modified composites. This may be the result of the presence of bulky lignin particles in the phenol matrix. Moreover, all modified samples had a lower glass transition temperature than the reference sample, which may be caused by the plasticizing properties of lignin [43,44], which can facilitate the preparation of resin blends and their processing during the formation of finished products. The lignin chains introduced into the matrix can increase the flexibility of the composites and may contribute to energy dissipation through internal friction [45]. The plasticizing effect of lignin-alumina fillers can decrease the fragility of composites. These phenomena may have a positive impact on the efficiency of the final abrasive tool [46].

### 2.7. Scanning Electron Microscopy Analysis of Composites

**Figure 11:**
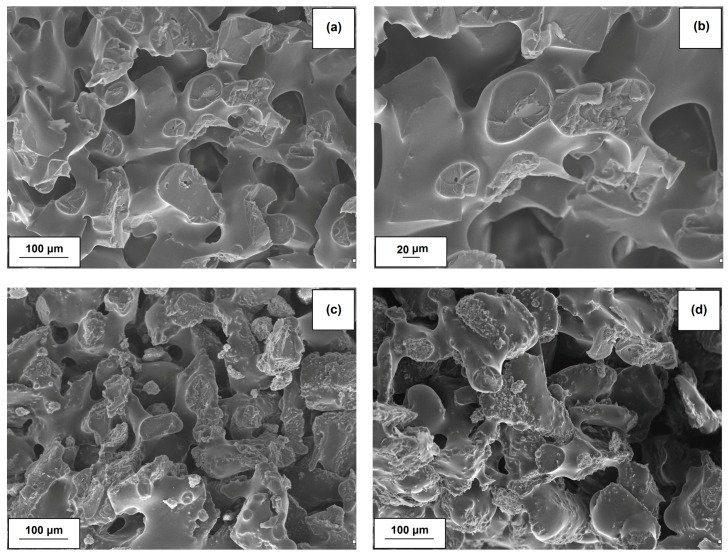
SEM images of novolac + corundum + resole composite (**a**,**b**) and novolac + corundum + resole + lignin-Al_2_O_3_ systems with ratios of organic-inorganic filler equal to 8:1 *wt*/*wt* (**c**) and 8:6 *wt*/*wt* (**d**).

The structure of composites with hybrid lignin-alumina fillers was fairly homogeneous. The abrasive grains were well-bounded in all of the studied fillers. Only some small filler agglomerates can be seen. There were no essential differences in the homogeneity of the composites depending on the ratio of lignin to alumina in the fillers. Particularly noteworthy are the SEM images of the composite without the organic-inorganic hybrid filler, consisting exclusively of novolac, corundum and resole (Figure 11a,b). The characteristic structures shown in the images demonstrate the homogeneity of the resulting mixture. The addition of organic-inorganic materials with appropriate ratios of lignin to alumina did not significantly deteriorate the morphological and microstructural properties (Figure 11c,d). Only small differences arise from the variation in the quantity of biopolymer relative to inorganic material (Table 6).

### 2.8. Assessment of Emission of Phenol and Formaldehyde by Means of HS-GC Analysis

The emission of phenol and formaldehyde was measured by HS-GC analysis. The peak area values for each tested sample were compared, which allowed to determine the influence of hybrid filler addition on the amount of two main volatile organic compounds released from the mixture. The peak with retention time equal to 1.07 min was attributed to formaldehyde and another with retention time equal to 1.21 min was attributed to phenol.

The values of the peak area for emitted formaldehyde are presented in Table 7 and for phenol in Table 8. The amount of released formaldehyde is slightly lower for sample with hybrid lignin-Al_2_O_3_ than for sample with Kraft lignin only. Moreover, the composition with kraft lignin emitted slightly more formadehyde than samples with zeolite micro 20, pure resol or pure novolak. This results from the fact that the Kraft lignin in temperatures above 180 °C undergoes thermal decomposition and emits formaldehyde among others [36]. Generally, no significant impact of studied fillers addition on formaldehyde emission can be observed.

In case of phenol emission, addition of lignin-Al_2_O_3_ hybrid caused a significant decrease of the amount of released phenol in comparison to Kraft lignin or zeolite micro 20. Addition of all studied fillers notably decreased the emission of phenol by approximately 2–3 times and the highest decrease of phenol emission was observed for composition with lignin-Al_2_O_3_ hybrid.

## 3. Materials and Methods

### 3.1. Preparation of Novel Lignin-Al_2_O_3_ Hybrid Filler

The novel, functional lignin-Al_2_O_3_ hybrid materials were prepared by a mechanical method from commercial alumina (Sigma-Aldrich, St. Louis, MO, USA) and Kraft lignin (Sigma-Aldrich). Hybrid additives were produced using 8 parts by weight of lignin with 1, 2, 4 and 6 parts of Al_2_O_3_, respectively. To combine the Al_2_O_3_ and lignin, a mechanical process was used whereby the initial powders were ground and simultaneously mixed using a Pulverisette 6 Classic Line planetary ball mill (Fritsch, Idar-Oberstein, Germany). The vessel with the materials for grinding was placed eccentrically on the mill’s rotating base. The direction of rotation of the base is opposite to that of the vessel, with a speed ratio of 1:2. The three agate balls inside the vessel move due to the Coriolis force. To obtain suitably homogeneous final materials, grinding was continued for 6 h. To prevent possible overheating of the material due to continuous grinding, every 2 h the mill automatically switched off for 5 min, after which it began operating again. Immediately after grinding, the lignin-Al_2_O_3_ hybrid materials were sifted using a sieve with a mesh diameter of 40 μm.

### 3.2. Preparation of Abrasive Composites with Lignin-Al_2_O_3_ Hybrids

The model abrasive composites were prepared by mixing resole, filler, novolac and abrasive grains, in a ratio of 3:5:12:80 by weight. The proportions of the components were chosen as the standard values used in the abrasive industry. The components were mixed using a mechanical mixer at a slow rate of 200 rpm for a short time (about 3 min)—the process was carried out at room temperature. White fused alumina with a 120 mesh granulation was used as an abrasive. Novolac contains 9% hexamethylenetetramine (hexamine). Firstly, the abrasive grains were covered by resole, then the mixture of novolac and filler was added and homogenized. The composites prepared this way were formed into cuboids. The samples were then hardened according to the following temperature program: heating from 50 °C up to 180 °C, heating rate 0.2 °C/min, then heating at 180 °C for 10 h.

### 3.3. Physicochemical and Dispersive-Morphological Characteristics of Lignin-Alumina Hybrids

#### 3.3.1. Particle Size Distribution

The dispersive properties of the products were evaluated using Mastersizer 2000 (0.2–2000 μm) and Zetasizer Nano ZS (0.6–6000 nm) instruments (Malvern Instruments Ltd., Malvern, UK), employing the laser diffraction and non-invasive back scattering (NIBS) techniques respectively. During the experiments, no pre-treatment was used for breaking down the agglomerates of the investigated products.

#### 3.3.2. Scanning Electron Microscopy

The surface morphology and microstructure of the lignin-alumina products and precursors were examined on the basis of SEM images recorded by an EVO40 scanning electron microscope (Zeiss, Jena, Germany). Before testing, the samples were coated with Au for a time of 5 s using a PV205P coater (Oerlikon Balzers Coating SA, Brügg, Switzerland).

#### 3.3.3. Fourier Transform Infrared Spectroscopy

Fourier transform infrared spectroscopy (FTIR) measurements were performed on a Vertex 70 spectrophotometer (Bruker, Mannheim, Germany) at room temperature (RT). The sample was analyzed in the form of pellets, made by pressing a mixture of anhydrous KBr (approximately 0.25 g) and 1.5 mg of the tested substance in a special steel ring under a pressure of approximately 10 MPa. FTIR spectra were obtained in the transmission mode between 4000 and 450 cm^−1^. The analysis was performed at a resolution of 0.5 cm^−1^.

#### 3.3.4. Thermogravimetric Analysis—Mass Spectrometry

TG-DSC analysis was carried out using a Jupiter STA 449 F1 instrument (Netzsch, Selb, Germany). The analysis was performed with a heating rate of 10 °C/min and a maximum temperature of 1000 °C. Measurements were conducted under a constant flow of helium (40 cm^3^/min). The sample mass was approximately 30 mg. The volatile products evolved during heating were detected by a 403C Aëolos mass spectrometer (QMS, Selb, Germany) coupled online to the STA instrument. The QMS was operated with an electron impact ionizer with an energy of 70 eV. During the measurements, the *m*/*z* ratio was recorded in the range of 2–150 amu, where *m* is the mass of the molecule and *z* its charge.

#### 3.3.5. Inverse Gas Chromatography

Surface properties of the hybrid fillers as well as alumina and lignin were tested by inverse gas chromatography (IGC). IGC experiments were carried out using a SEA Advanced apparatus (Surface Energy Analyzer produced by Surface Measurement System Ltd., London, UK) equipped with a flame ionization detector. The studied hybrid fillers were applied to inert glass beads in a quantity of 1% (200 mg), placed in a glass chromatographic column (30 cm length, 0.4 cm inner diameter). The column oven temperature was 30 °C, and the temperature of the detector and injector was 150 °C. Dead time was determined by means of methane injection. Helium (flow rate 15 cm^3^/min) was used as the carrier gas. The following test compounds were used: nonpolar—hexane, heptane, octane, nonane, decane; and polar—ethyl acetate, dichloromethane, ethanol, dioxane, acetonitrile, acetone.

The free surface energy, $\gamma_{s}^{total}$, and its dispersive ($\gamma_{s}^{d}$) and specific components (acid, $\gamma_{s}^{+}$ and basic, $\gamma_{s}^{-}$) were determined. The $\gamma_{s}^{d}$ parameter was calculated according to the Schultz–Lavielle method, using Equation (1) [47]:

(1) $$R\cdot T\cdot \ln V_{N}=2\cdot N\cdot a\cdot \sqrt{\gamma_{s}^{d}\cdot \gamma_{l}^{d}}+C$$

where: *R* is the gas constant, 8.314 J/mol·K; *T* is the temperature of measurement (K); $V_{N}$ is the net retention volume (m^3^); *N* is Avogadro’s constant, 6.022 × 10^23^ L/mol; *a* is the cross-sectional area of the adsorbate (m^2^); $\gamma_{s}^{d}$ is the dispersive component of surface free energy (mJ/m^2^); $\gamma_{l}^{d}$ is the dispersive component of the surface tension of the probe molecule in liquid state (mJ/m^2^); *C* is a constant.

Retention data for polar and nonpolar test compounds are necessary to quantify the acidic and basic properties of the examined surface. These are described by the parameters $\gamma_{s}^{+}$, $\gamma_{s}^{-}$, which were estimated according to the Good–van Oss concept [48] described by Equation (2):

(2) $$∆G^{sp}=2\cdot N_{A}\cdot a\cdot ({\left(\gamma_{l}^{+}\cdot \gamma_{s}^{-}\right)}^{1/2}+{\left(\gamma_{l}^{-}\cdot \gamma_{s}^{+}\right)}^{1/2})$$

In Equation (2), $\gamma_{l}^{+}$, $\gamma_{l}^{-}$ are the electron acceptor and donor parameters of the probe molecules, respectively, and $∆G^{sp}$ is the specific component of the free energy of adsorption of the polar compound. The method of determination of Δ*G^sp^* is described in many publications, e.g., [47,48]. For the calculation of $\gamma_{s}^{+}$, $\gamma_{s}^{-}$ dichloromethane (DM) and ethyl acetate (EA) were used as test compounds. DM is a monopolar acid, and $\gamma_{DM}^{-}$ = 0.0 mJ/m^2^. Equation (2) is reduced to:

(3) $$\gamma_{S}^{-}=∆G_{DM}^{sp}/(4\cdot N_{A}{}^{2}\cdot a_{DM}^{2}\cdot \gamma_{DM}^{+})$$

and $\gamma_{S}^{-}$ can be easily calculated. The value of $\gamma_{DM}^{+}$ was established as 5.2 mJ/m^2^ on the basis of [48]. Similarly, EA is a monopolar base, $\gamma_{EA}^{+}$ = 0.0 mJ/m^2^, and the $\gamma_{S}^{+}$ parameter for the examined solid can be calculated from Equation (4):

(4) $$\gamma_{S}^{+}=∆G_{EA}^{sp}/\left(4\cdot N_{A}{}^{2}\cdot a_{EA}^{2}\cdot \gamma_{EA}^{-}\right)$$

The value of $\gamma_{EA}^{-}$ was established as 19.2 mJ/m^2^ [46].

The acid-base properties of the studied fillers, as well as lignin and alumina, were assessed in terms of the parameters *K_A_* and *K_D_*, describing respectively the acid and base properties of the surface. These parameters were calculated from the straight line (Equation (5)):

(5) $$\frac{\Delta G_{sp}}{AN_{}^{*}}=K_{A}\cdot \frac{DN_{}}{AN_{}^{*}}+K_{D}$$

where: *K_A_* is the parameter expressing the acidic properties of the solid surface; *K_D_* is the parameter expressing the basic properties of the solid surface; Δ*G_sp_* is the specific component of the free energy of adsorption of the polar compound; *DN* is the donor number of the polar test solute; AN* is the acceptor number of the polar test solute.

### 3.4. Rheological Studies

Samples for rheological measurements were prepared by a mechanical method in a closed container with simultaneous mixing. The following composites were studied: novolac and lignin; novolac and Al_2_O_3_; novolac and lignin-Al_2_O_3_ hybrid (8:1, 8:2, 8:4 and 8:6 *wt*/*wt*).

Pure novolac was measured as a reference sample, as well as novolac with additive (constant proportion 3:1.25 *wt*/*wt*). To measure samples in a powder state in the rotational rheometer, it was decided to divide the measurements into two steps: firstly sample preparation, and secondly monitoring of the curing process. The rheological behavior of a sample was tested using an RS6000 Thermo Scientific rheometer (HAAKE, Vreden, Germany) with disposable plate-plate rotor with diameter 20 mm and disposable lower plate. The disposable measuring system enables monitoring of the cross-linking of the resin up to total curing.

The rheometer was set to an initial temperature of 80 °C. Next, the sample was loaded onto the rheometer with a sample loading tool. With the border around the lower plate, the geometry can easily be filled with powders. Next, the automatic lift, controlled by a RheoWin device (HAAKE), moves the upper geometry into the measuring position with a force of 20 N for 90 s. After this procedure, the sample forms a puck with the same geometry, independently of the bulk density. The measuring position was reached when the rheometer touched the sample with a normal force of 5 N. Next, a temperature module with the Peltier system started to change the temperature of the sample from 80 to 100 °C over 30 min. This process allowed to soften the sample.

In the next step, the sample prepared as described above was cross-linked in a temperature sweep from 100 to 160 °C over 30 min, and the cross-linking process was further monitored at 160 °C for 15 min. For protection against heating loss, a solvent trap made from teflon was used. Because in the first step of the sample preparation process the samples were observed to have different structures, the rheometer achieved a gap of 1.2 mm. When the rheometer rotor moved down to the measuring position, the normal force was recorded. Attainment of the normal force can be characterized by the degree of softening of the sample and its thermal conductivity. The classical rheological measurement to monitor the curing process is the oscillation test. The oscillation test is nondestructive when a small deformation or stress is involved. Because the viscoelastic properties of the sample vary over a large range in the curing process, the method with controlled deformation γ = 0.01 was chosen. For characterization of viscoelastic properties, storage modulus represents the elastic nature of the sample, while the loss modulus represents its viscous nature. When the sample forms a network structure in the curing process, it is expected that changes in the nature of the sample from more viscous to more elastic will be observed. Equilibrium of elastic properties means that a full cure state was reached in the curing process.

### 3.5. Dynamic-Mechanical Properties

The dynamic-mechanical properties of samples with dimensions of 10 × 4 × 50 mm were investigated by dynamic mechanical thermal analysis (DMTA) in torsion mode using an Anton Paar MCR 301 apparatus (Ashland, VA, USA) operating at a frequency of 1 Hz. The temperature range was from 25 to 300 °C, with a heating rate of 2 °C/min. The position of tan δ at its maximum was taken as the glass transition temperature.

### 3.6. Headspace Gas Chromatography

An automatic headspace sampler (TurboMatrix HS 40, PerkinElmer Waltham, MA, USA) and a gas chromatograph system (Clarus 580, PerkinElmer) were used for HS-GC measurement. The GC system was equipped with a flame ionization detector and an Elite-5 capillary column (30 m × 0.25 mm i.d., with 0.25 μm film thickness, PerkinElmer) operating at temperatures of: vial 180 °C, transfer line 200 °C, column 210 °C with helium transfer gas (flow rate 2 mL/min) was employed. All examined samples consisted of: 0.95 g of organic resin binder (MD 1/11 novolak resin, LERG S. A., Pustków-Osiedle, Poland), 0.2 g of wetting agent (Rezol S resole resin, LERG S. A., Pustków-Osiedle, Poland) and 0.35 g of tested filler (pure lignin or lignin-Al_2_O_3_ hybrid (8:4 *wt*/*wt*) as well as zeolite micro 20—filler commercially used in abrasive industry). Also the samples of: (i) 0.35 g Kraft lignin; (ii) 0.95 g novolak and (iii) 0.2 g Rezol S were tested. All the components were precisely mixed together to achieve a homogeneous mixture. The vial thermostating time was equal to 5 min. The number of injections for each sample was equal to 7: the analysis was performed 7 times for the same vial as multiple headspace till the peak area decreased significantly (near the limit of detection). The repetition of the method was determined by performance of the analysis for the same composition three times by preparing new vial as described above. The volume of headspace vials was 20 mL. The qualitative analysis of phenol and formaldehyde was performed on the basis of comparison of the retention time of the tested materials with standards: pure phenol and formaldehyde (paraformaldehyde). Phenol and formaldehyde were of analytical grade (purity > 99%, Sigma Aldrich, Steinheim am Albuch, Germany). Each compound was placed in the vial separately and the HS-GC analysis was performed as for other studied materials.

## 4. Conclusions

The results presented in the framework of this study demonstrate that novel lignin-alumina hybrid fillers, which were not previously described in the literature, can be obtained in a relatively simple way by intensive mechanical mixing of the biopolymer with Al_2_O_3_. The use of lignin-alumina hybrids makes it possible to obtain final composite abrasive articles with higher plasticity due to the lignin part, and also better heat conductivity due to the Al_2_O_3_. Moreover, it turns out that the addition of even a small quantity of alumina (lignin-to-alumina ratio 8:1 *wt*/*wt*) can increase the thermal conductivity of lignin, and thus improve the thermomechanical properties of the final composite used for abrasive tool production. The inorganic-organic hybrid fillers added to the composition of the abrasive tool have the most influence on the dynamics of cross-linking at temperatures of approx. 160 °C and the “internal” turbulence process at temperatures of approx. 140 °C, by changing the normal force. It is worth noticing that the addition of lignin-Al_2_O_3_ hybrids notably decreased phenol emission and slightly limited formaldehyde emission in comparison to commercially used filler natural zeolite micro 20 as well as pure Kraft lignin. The thorough physicochemical analysis of the new hybrid fillers has shown that chemical bonds may be formed between the hydroxyl groups present in both lignin and alumina. Further research will certainly be continued in this direction, especially with the use of kraft lignin derivatives combined with alumina using mechanical and chemical methods to increase the interaction between the precursors. Additionally, the study of the durability properties of the adhesives, using natural and/or QUV accelerated tests to prevent the ageing effects of temperature, humidity and UV exposure on the coating will be particularly important in the near future.

## Figures and Tables

**Table 1:** Dispersive characteristic of pure alumina and lignin-alumina hybrid fillers.

Sample	Dispersive Properties				
	Particle Size Distribution from Zetasizer Nano ZS (nm)	Particle Diameter from Mastersizer 2000 (µm)			
		d(0.1) *	d(0.5) **	d(0.9) ***	D(4.3) ****
Al_2_O_3_	142–955	2.5	3.7	5.5	3.7
Lignin-Al_2_O_3_ (8:1, *wt*/*wt*)	531–1106	2.1	3.6	5.3	3.3
Lignin-Al_2_O_3_ (8:2, *wt*/*wt*)	396–825	2.5	3.7	5.2	3.4
Lignin-Al_2_O_3_ (8:4, *wt*/*wt*)	295–955	2.4	3.5	5.2	3.2
Lignin-Al_2_O_3_ (8:6, *wt*/*wt*)	142–825	2.4	3.6	5.3	3.4

* d(0.1)—10% of the volume distribution is below this diameter value; ** d(0.5)—50% of the volume distribution is below this diameter value; *** d(0.9)—90% of the volume distribution is below this diameter value; **** D(4.3)—average particle size in examined system.

**Table 2:** Vibrational frequency wavenumbers (cm^−1^) for lignin, Al_2_O_3_ and lignin-Al_2_O_3_ fillers.

Lignin	Alumina	Lignin-Al_2_O_3_ Hybrid	Vibrational Assignment
-	3635, 3543 and 3473	overshadowed	Al–OH stretching
3432	3145	3430	O–H stretching, absorbed water
2935, 2877	-	2937, 2879	CH*_x_* stretching
1648	-	1646	C=O stretching
1618	1620	1619	O–H bending of water
1602 not visible	-	1602 not visible	C–C, C=C (aromatic skeleton), stretching
1508	-	1508	
1471	-	1470	C–H (CH_3_ + CH_2_), bending
1419	-	1418	C–C, C=C (aromatic skeleton), stretching
-	1390	1389	Al–O as Si cage (TO_4_)
1271	-	1271	C–O (guaiacyl unit) stretching
1226	-	1226	C–OH (phenolic OH) stretching
1139	-	1140	Aromatic C–H (guaiacyl unit), stretching
1080	-	1077	C–O stretching
1045	-	1039	C–OH + C–O–C (aliphatic OH + ether) stretching
-	1035	1039	Al–OH symmetric bending
-	970, 893	969, 893	–OH deformation linked to Al^3−^
856, 751	-	858, 751	Aromatic C–H (guaiacyl unit), bending
-	788, 750, 693, 564 and 512	788, 751, 695, 565 and 512	Al–O in which aluminum ions are in both tetrahedral and octahedral sites
534	-	534	CH*_x_* bending

**Table 3:** Comparison of lignin and different lignin-Al_2_O_3_ composition collected during thermal decomposition of samples. DTG and Tp are first derivative of sample mass loss signal and peek temperature, respectively.

Sample Composition	Tonset from DSC (°C)			
**Lignin**	**Lignin-Al_2_O_3_ Hybrid Fillers (*wt*/*wt*)**			
	**8:1**	**8:2**	**8:4**	**8:6**
34	32	27	28	28
324	330	329	328	327
650	645	678	646	640
**Speed of Decomposition from DTG (%/min)**				
**Lignin**	**Lignin-Al_2_O_3_ Hybrid Fillers**			
	**8:1**	**8:2**	**8:4**	**8:6**
0.85	0.72	0.69	0.50	0.58
2.14	1.98	1.76	1.43	1.23
0.65	0.72	0.70	0.71	0.20
**Sample Mass Loss (%), Where Temperature Ranges Are, a: RT-160 °C, b: 160–550 °C, c: 550–1000 °C**				
6.8 ^a^	5.9 ^a^	5.6 ^a^	4.4 ^a^	4.1 ^a^
33.8 ^b^	31.0 ^b^	28.1 ^b^	23.6 ^b^	20.4 ^b^
20.2 ^c^	13.2 ^c^	13.0 ^c^	12.2 ^c^	9.2 ^c^

**Table 4:** Dispersive, ($\gamma_{s}^{d}$) donor-acceptor (γ+, γ−) components of the free surface energy of studied hybrid fillers and comparison with alumina and lignin.

Sample	$\gamma_{s}^{d}$ (mJ/m^2^)	$\gamma_{}^{+}$ (mJ/m^2^)	$\gamma_{}^{-}$ (mJ/m^2^)	*K_A_* (-)	*K_D_* (-)	*K_A_*/*K_D_* (-)
Al_2_O_3_	37.2 ± 0.5	29.0 ± 0.1	177.2 ± 5.0	0.100 ± 0.010	0.260 ± 0.003	0.385
Lignin	35.2 ± 0.6	15.2 ± 0.2	46.4 ± 1.0	0.112 ± 0.005	0.161 ± 0.002	0.702
Lignin-Al_2_O_3_ (8:1, *wt*/*wt*)	33.2 ± 0.4	11.5 ± 0.1	38.2 ± 1.1	0.071 ± 0.001	0.140 ± 0.004	0.507
Lignin-Al_2_O_3_ (8:2, *wt*/*wt*)	36.4 ± 0.3	9.8 ± 0.2	35.8 ± 0.8	0.067 ± 0.001	0.143 ± 0.007	0.469
Lignin-Al_2_O_3_ (8:4, *wt*/*wt*)	35.6 ± 0.1	10.5 ± 0.1	39.3 ± 0.5	0.068 ± 0.001	0.146 ± 0.003	0.466
Lignin-Al_2_O_3_ (8:6, *wt*/*wt*)	35.7 ± 0.1	27.1 ± 0.3	148.1 ± 3.0	0.101 ± 0.002	0.220 ± 0.003	0.459

**Table 5:** Characteristic points for the curing process.

Sample	Softening Point		Cross Over Point	
	Temperature (°C)	Viscosity (Pa·s)	Temperature (°C)	G′ = G″ (Pa)
Al_2_O_3_	136.4	1.029	158	27,400
Lignin	136.1	49.68	157	898,000
Lignin-Al_2_O_3_ (8:1, *wt*/*wt*)	135.5	3950	158	430,000
Lignin-Al_2_O_3_ (8:2, *wt*/*wt*)	135.7	2913	160	533,000
Lignin-Al_2_O_3_ (8:4, *wt*/*wt*)	136.7	1294	158	680,000
Lignin-Al_2_O_3_ (8:6, *wt*/*wt*)	135.6	633	160	326,000

**Table 6:** Values of storage modules and glass transition temperature of composites obtained.

Sample	G′ 25 °C (MPa)	G′ 50 °C (MPa)	G′ 300 °C (MPa)	Tan δ_max_	T_g_ (°C)
Reference sample	2750	2680	1350	0.055	252
Lignin-Al_2_O_3_ (8:1, *wt*/*wt*)	1690	1640	776	0.076	244
Lignin-Al_2_O_3_ (8:2, *wt*/*wt*)	1150	1110	530	0.067	250
Lignin-Al_2_O_3_ (8:4, *wt*/*wt*)	1520	1470	691	0.068	247
Lignin-Al_2_O_3_ (8:6, *wt*/*wt*)	1470	1420	652	0.071	244

**Table 7:** The value of peak area of the formaldehyde emitted from tested samples during HS analysis.

Sample	Peak Area, *S_peak_* (µV·s) *
Novolac	1.21 × 10^6^ ± 0.11 × 10^6^
Resol	1.90 × 10^6^ ± 0.28 × 10^6^
Kraft lignin	2.52 × 10^6^ ± 0.09 × 10^6^
Lignin-Al_2_O_3_ (8:4, *wt*/*wt*)	2.30 × 10^6^ ± 0.08 × 10^6^
Resol + novolac + Kraft lignin	2.73 × 10^6^ ± 0.18 × 10^6^
Resol + novolac + lignin-Al_2_O_3_ (8:4, *wt*/*wt*)	2.24 × 10^6^ ± 0.18 × 10^6^
Resol + novolac + zeolite micro 20	2.05 × 10^6^ ± 0.20 × 10^6^

* The sum of seven injections from the same vial ± standard deviation for three repetitions of the whole analysis for three independent vials.

**Table 8:** The value of peak area of the phenol emitted from tested samples during HS analysis.

Sample	The Peak Area, *S_peak_* (µV·s) *
Novolac	0.32 × 10^6^ ± 0.05 × 10^6^
Resol	10.53 × 10^6^ ± 0.60 × 10^6^
Kraft lignin	0.02 × 10^6^ ± 0.00 × 10^6^
Lignin-Al_2_O_3_ (8:4, *wt*/*wt*)	0.01 × 10^6^ ± 0.00 × 10^6^
Resol + novolac + Kraft lignin	5.49 × 10^6^ ± 0.40 × 10^6^
Resol + novolac + lignin-Al_2_O_3_ (8:4, *wt*/*wt*)	3.47 × 10^6^ ± 0.31 × 10^6^
Resol + novolac + zeolite micro 20	4.86 × 10^6^ ± 0.45 × 10^6^

* The sum of seven injections from the same vial ± standard deviation for three repetitions of the whole analysis for three independent vials.
